# *MSH1*-Induced Non-Genetic Variation Provides a Source of Phenotypic Diversity in *Sorghum bicolor*

**DOI:** 10.1371/journal.pone.0108407

**Published:** 2014-10-27

**Authors:** Roberto de la Rosa Santamaria, Mon-Ray Shao, Guomei Wang, David O. Nino-Liu, Hardik Kundariya, Yashitola Wamboldt, Ismail Dweikat, Sally A. Mackenzie

**Affiliations:** 1 Center for Plant Science Innovation, University of Nebraska Lincoln, Lincoln, Nebraska, United States of America; 2 Department of Agronomy and Horticulture, University of Nebraska Lincoln, Lincoln, Nebraska, United States of America; 3 Monsanto, Chesterfield Village Research Center, Chesterfield, Missouri, United States of America; Agriculture and Agri-Food Canada, Canada

## Abstract

*MutS Homolog 1* (*MSH1*) encodes a plant-specific protein that functions in mitochondria and chloroplasts. We showed previously that disruption or suppression of the *MSH1* gene results in a process of developmental reprogramming that is heritable and non-genetic in subsequent generations. In Arabidopsis, this developmental reprogramming process is accompanied by striking changes in gene expression of organellar and stress response genes. This developmentally reprogrammed state, when used in crossing, results in a range of variation for plant growth potential. Here we investigate the implications of *MSH1* modulation in a crop species. We found that *MSH1*-mediated phenotypic variation in *Sorghum bicolor* is heritable and potentially valuable for crop breeding. We observed phenotypic variation for grain yield, plant height, flowering time, panicle architecture, and above-ground biomass. Focusing on grain yield and plant height, we found some lines that appeared to respond to selection. Based on amenability of this system to implementation in a range of crops, and the scope of phenotypic variation that is derived, our results suggest that *MSH1* suppression provides a novel approach for breeding in crops.

## Introduction

One increasingly problematic threat to plant improvement is the depletion of natural stores of genetic diversity for most of our major crop species [1]. Centers of diversity for many species have been encroached by man-made or natural influences, limiting our ability to diversify germplasm appropriate for breeding efforts. Moreover, integration of unselected germplasm to a breeding program is laborious in the early selection process required to eliminate undesirable genetic linkages [2].

For years, breeders have speculated that non-genetic variation plays a key role in conventional crop improvement strategies [3]. For example, epigenetic influences have been implicated in heterosis [4], [5], flowering time and maturation [6], [7], inbreeding depression and its circumvention [8], and genotype×environmental interactions [9], [10]. Still, there has been no straightforward means of directly accessing such variation for plant improvement purposes.

*MSH1* is a plant-specific gene that encodes a mitochondrial and plastid-localized protein [11], [12]. The expression level of *MSH1* appears to be influenced by environmental stress [12], [13]. In Arabidopsis, *msh1* mutants are characterized by variable phenotypes including dwarfing, variegation, delay in maturity transition and flowering, altered branching, and woody growth with aerial rosettes at short day length growth [14]. This developmental reprogramming (MSH1-dr) is associated with large changes in gene expression, particularly genes involved in organelle and stress response functions [14]. RNAi suppression of *MSH1* in crop plants, including tomato, soybean, tobacco, millet and sorghum produces a similar MSH1-dr phenotypic range in each that is subsequently inherited independent of the RNAi transgene [14]. These observations suggest that the MSH1-dr phenotype is both programmed and non-genetic.

Here we investigate the consequences of incorporating the MSH1-dr condition to plant selection, using sorghum as a model. We show that crossing with a transgene-null MSH1-dr line produces an unexpected range of phenotypic variation that is both heritable and responsive to selection. This variation appears to be stable over at least four generations. We also show evidence of line×environment interactions. Finally, we demonstrate gains in grain yield over two generations of selection, suggesting that this non-genetic variation may prove valuable for agricultural production as a potential crop breeding strategy.

## Materials and Methods

### Plant materials and growth conditions

Sorghum MSH1-dr plants used in these experiments were derived as described in [14]. Six T_3_ individuals displaying the MSH1-dr phenotype but null for the MSH1-RNAi transgene were used as females in crosses to wild type inbred Tx430 to derive F_1_ seed. Another three T_3_ individuals were used as males in the reciprocal crosses to Tx430. The number of F_1_ plants derived from each cross ranged from 5 to 19 individuals. Parents and F_1_ progeny were grown under greenhouse conditions on a 14 hr/10 hr day-night cycle with 28°C/22°C day-night temperatures. Self-pollinated seed of F_1_ plants was harvested individually to generate corresponding F_2_ families.

### Field experiments and phenotyping

In all field plots, plants were thinned to a final density of 15 plants/m^2^ and fertilized according to standard growing practices. The 2010 field experiment was used to propagate F_2_ lines, and contained F_2_ and wild type Tx430. The 2011 field experiment contained F_2_, F_3_, and F_4_ lines randomized across seven blocks with 28 rows per block (alpha lattice design) and two field replicates. Replicates were augmented with wild type Tx430 (16 rows total).

For estimating grain yield, threshed panicles from three plants were pooled and converted to grams/m^2^ based on final plant density, with 2–3 such measurements taken per row. For comparison of panicle yield distributions in F_2_ versus in wild type Tx430, individual panicle grain yield (i.e., prior to pooling) was used. For flowering time, plant height, and rachis length, measurements were taken on individual plants. For each dry biomass measurement, three fully dried plants were pooled together then converted to grams/plant. Plants showing the DR phenotype were not included in phenotypic variation analysis.

The 2012 multi-location experiment included Lincoln, NE (40° 51′N, 96° 35′W) and Mead, NE (41° 9′N, 96° 24′W) sites, which received 178 mm and 158 mm of precipitation over the growing season, respectively. Within each location, lines were grown in two-row plots arranged in a randomized complete block design with two replicates. For this experiment, grain yield was estimated by taking threshed panicles from a meter-length area of each row and converting to grams/m^2^.

### Statistical analysis

For evaluations in a single environment, mean phenotypic values and confidence intervals for each line were estimated using the linear mixed model *y_ijk_* = *μ*+*α_i_*+*r_k_*+(*b/r*)*_jk_*+*ε_ijk_* where *y_ijk_* is the trait response, *μ* is the population mean, *α_i_* is the effect of line *i*, *r_k_* is the effect of replicate *k*, (*b/r*)*_jk_* is the effect of block *j* nested within replicate *k*, and *ε_ijk_* is the residual error. For evaluations over multiple environments, mean phenotypic values and confidence intervals for each line were estimated using the linear mixed model *y_ijkm_* = *μ*+*α_i_*+*e_m_*+(*r/e*)*_km_*+(*b/r/e*)*_jkm_*+(*αe)_im_*+*ε_ijkm_* where *y_ijkm_* is the trait response, *μ* is the population mean, *α_i_* is the effect of line *i*, *e_m_* is the effect of environment *m*, (*r/e*)*_km_* is the effect of replicate *k* nested within environment *m*, (*b/r/e*)*_jkm_* is the effect of block *j* nested within replicate *k* of environment *m*, (*αe)_im_* is the interaction between line *i* and environment *m*, and *ε_ijkm_* is the residual. Line, environment, and line×environment effects were treated as fixed while block and replicate effects were treated as random. Models were fit by restricted maximum likelihood using the R package “nlme” [15]. When deemed appropriate, Box-cox transformations were performed. F_4_ models for plant height and biomass excluded lines exhibiting mixed heights to avoid heteroscedasticity.

### PCR assay for RNAi transgene and SSR marker analysis

PCR assay for MSH1-RNAi transgene presence in sorghum materials used primers RNAi-F 5′-GTGTACT CATCTGGATCTGTATTG-3′ and RNAi-R 5′-GGTTGAGGAGCCTGAATCTCTGAAC-3′. Positive and negative controls were included from a confirmed transgenic line and wild type Tx430, respectively.

SSR marker analysis used SSR primers that were developed and mapped previously [16], [17]. Fragments were assayed by capillary electrophoresis on an Advanced Analytical Fragment Analyzer (Advanced Analytical Technologies, Inc. Ames, IA) using the dsDNA Reagent kit, 35–1,500 bp 500S that separates DNA in the size range of 35–1,500 bp. Of the 136 primers that were tested, 43 produced unambiguous polymorphisms between Tx430 and the sweet sorghum control line Wray and were used for testing the epi-lines.

### Sorghum SNP survey

Leaf tissue sample was collected from plants grown under controlled greenhouse conditions three weeks after germination. Genomic DNA was extracted from freeze-dried leaf tissue and processed following manufacturer's recommendations prior to Infinium beadchip hybridization (Illumina, San Diego, CA). The genotyping of five F_4_ lines and wild type Tx430 was carried out at the Monsanto Applied Genotyping Labs (Chesterfield, MO). The platform used was an exclusive custom-designed *Sorghum bicolor* Infinium high-density beadchip containing 1,885 internally validated SNP markers.

For the six samples, 107 of the 1,885 SNP markers, ca 5.68%, provided invalid data due to one of the following: low marker signal intensity, marker failed data QC, or unscorable allele calls. The remaining 1,778 SNP markers were used for the analysis. These 1,778 SNP markers are distributed across all 10 sorghum chromosomes with genome coverage approximating 90%. The number of heterozygotes (# Het) and percentage of heterozygotes (% Het) were calculated based on the 1778 SNP markers.

## Results

### *MSH1*-altered lines and reciprocal crosses

Previously, we described MSH1-RNAi lines displaying numerous physiological changes, a condition of developmental reprogramming that was termed MSH1-dr [14]. Segregation of the MSH1-RNAi transgene gave rise to some *MSH1* +/+ individuals that retained the characteristic *msh1* phenotype despite having normal *MSH1* transcript levels [14]. These plants maintain the altered MSH1-dr growth phenotype through multiple (at least nine, to date) generations of self-pollination.

**Figure 1:**
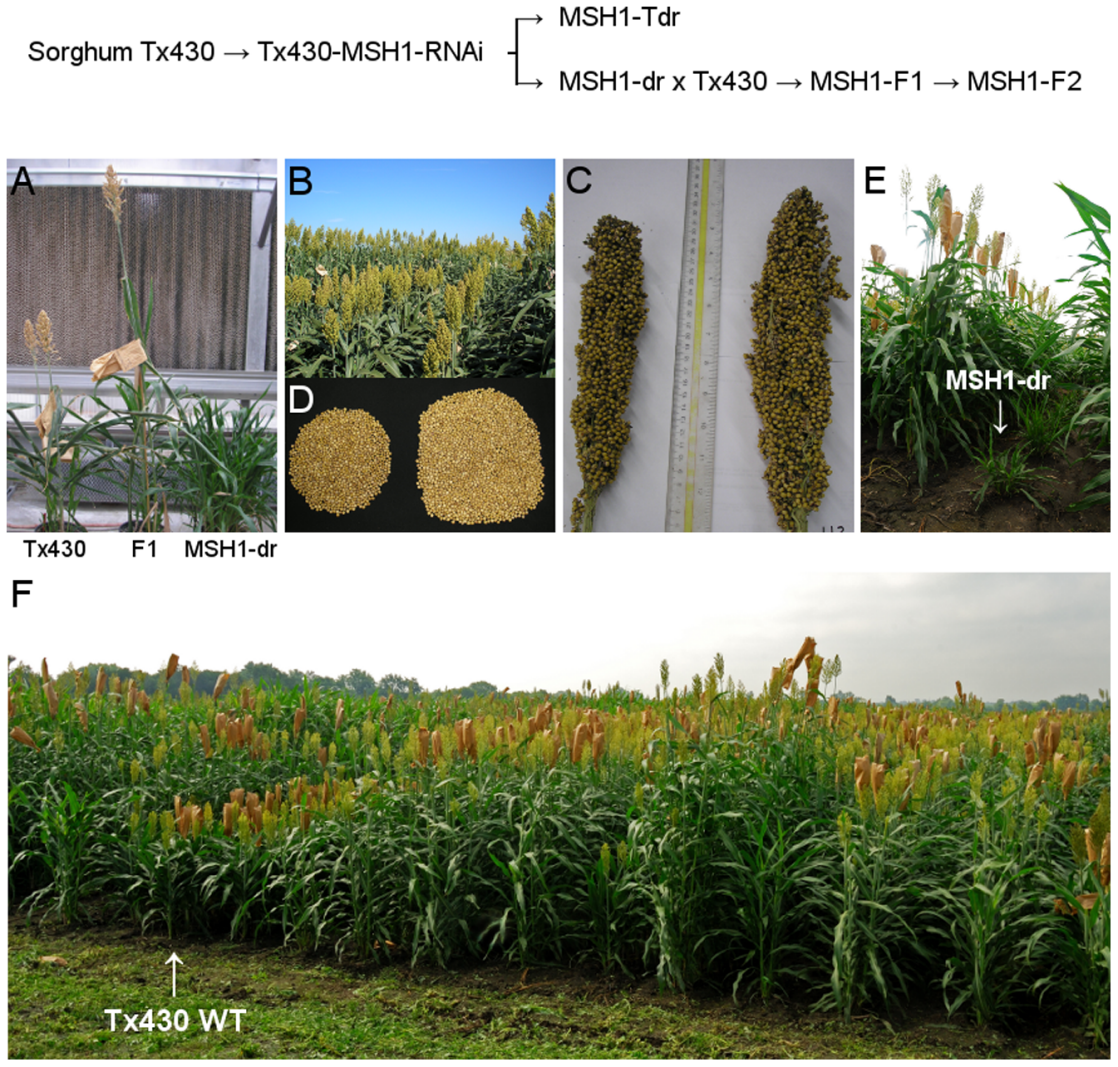
Enhanced growth phenotype of MSH1 lines in sorghum. The transgene and crossing procedure used to derive sorghum populations is indicated. (A) The phenotype of the F_1_ progeny derived from crossing Tx430×MSH1-dr. (B) Field grown F_2_, F_3_ and F_4_ sorghum lines show variation in plant architecture and height. (C) Panicles from Tx430 (on left, 66 g, 8 mm stem) versus a larger F_2_ individual (on right, 112 g, 11 mm stem), and (D) seed yield after threshing. (E) The MSH1-dr sorghum phenotype under field conditions. (F) Sorghum MSH1 F_2_, F_3_ and F_4_ populations grown in progeny rows in a 2011 field experiment. Wild type inbred Tx430 is indicated. Variation in plant height, flowering time and plant architecture is apparent; all plants shown are non-transgenic and Tx430 genotype.

To investigate the mechanism of inheritance, we performed reciprocal crosses in sorghum of MSH1-dr individuals to their wild type counterpart. Figure 1 illustrates the transgene and crossing process used in this study, with all sorghum materials generated from the inbred line Tx430 [18]. When crossed to the wild type inbred Tx430 line, the transgene-null MSH1-dr individuals produced progeny that were restored to normal phenotype (Figure 1A). The derived F_1_ progeny no longer showed the dwarfed, tillering, and late flowering phenotype; instead, many of the plants grew taller and produced more seed than the wild type. This was repeatedly observed in F_1_ populations derived from nine separate crosses, three of which used an MSH1-dr plant as the pollen donor [14].

Lack of the MSH1-dr phenotype in the F_1_ generation from either direct or reciprocal crosses argues against the observed phenotypes in this sorghum material being inherited via cytoplasmic organellar genomes. Analogously generated crosses in Arabidopsis with *msh1* point or T-DNA insertion mutations also display enhanced vigor; in other species, including tomato, soybean and tobacco, heritable MSH1-dr phenotypes also persist despite restored *MSH1* expression following RNAi silencing, and crosses in those species to their respective wild type counterparts similarly produce progeny with enhanced growth phenotypes [14], (unpublished data). Taken together, the evidence suggests that the MSH1-dr and F_1_ observations involve a conserved, programmed pathway.

### MSH1 F_2_ populations show enhanced variation

**Figure 2:**
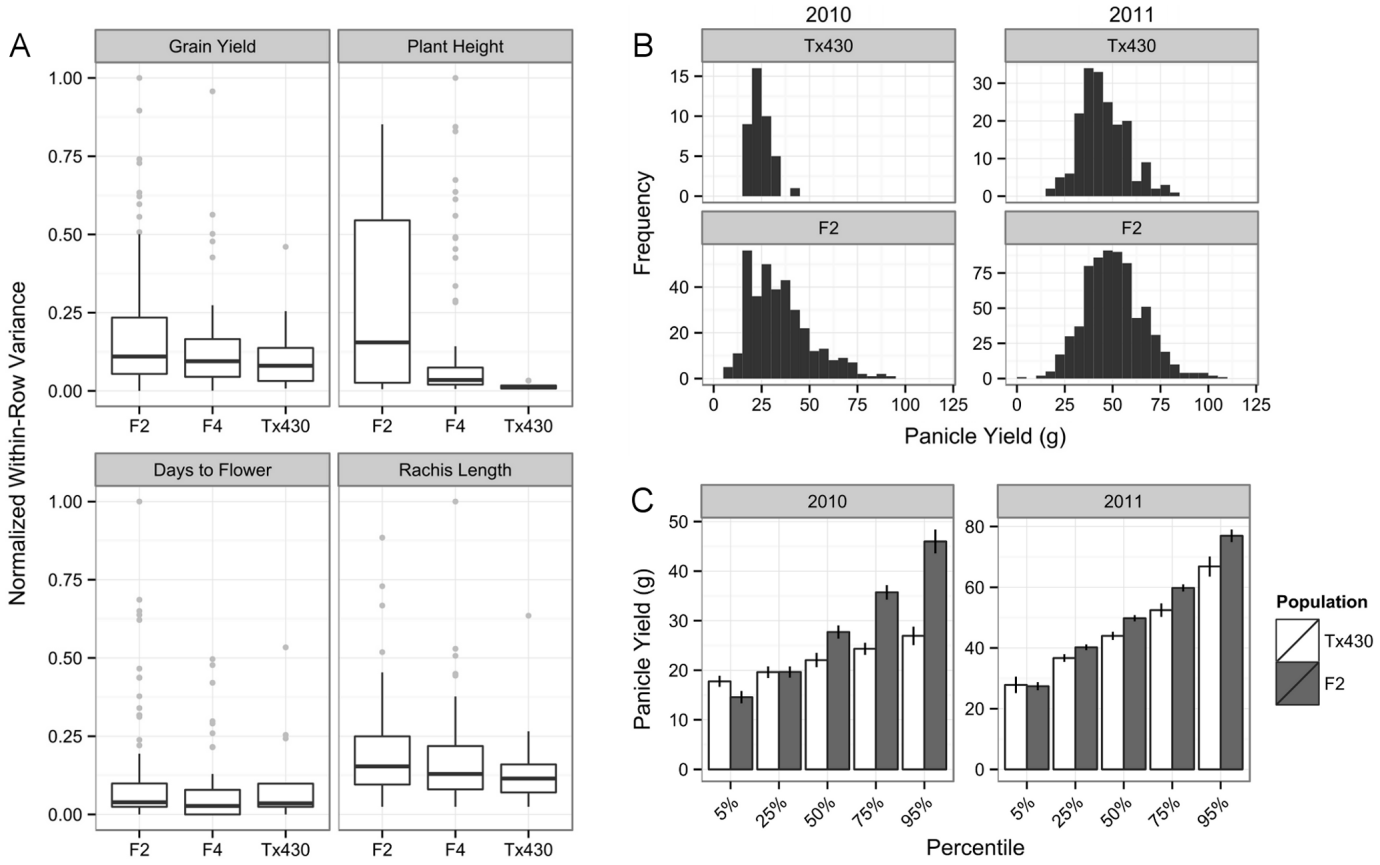
Increased phenotypic variation in MSH1 F2 lines. (A) Boxplots of within-row field variance for indicated traits, with values normalized as a proportion of the maximum observed row variance for that trait. Differences in variances between the F_2_ and wild type populations were significant for plant height (Brown-Forsythe test, *p*<0.001) and grain yield (*p*<0.01). (B) Histograms for yield per panicle in the F_2_ population compared to wild type, from the two field plantings. (C) Percentile values for yield per panicle in the F_2_ population compared to wild type, estimated from bootstrapping; error bars represent standard deviation.

Self-pollination of the F_1_ plants produced an F_2_ population variable in plant phenotype (Figure 1B–F, Figure 2, Table S1), with a minority exhibiting the MSH1-dr phenotype (Figure 1E). This was initially apparent in several F2 families as an elongated tail in the distributions for panicle weight, suggesting a higher proportion of individuals with extreme values (Figure S1). Further analysis detected increased variation in the F_2_ for plant height and grain yield (Figure 2A, Table S1), which although more prominent in the 2010 planting than the 2011 planting (Figure 2B–C), was still significant (Table S1). Although we did not detect a very significant increase in variance for flowering time or panicle length in the F_2_, by the F_4_ we were able to observe lines diverging from wild type Tx430 for those traits (Figure S2), suggesting modest but heritable variation for flowering time and panicle length.

A small proportion of greenhouse-grown MSH1 F_3_ families also showed the MSH1-dr phenotype, with an overall frequency of ca. 8% (Table S2). By the F_4_ generation, we estimate that the overall frequency drops to below 2%. Although the progeny from these sporadic MSH1-dr types in advanced generations have not been thoroughly investigated, some families appear more likely than others to produce this phenotype. When MSH1-dr frequencies were compared between parental and progeny generations, each derived from a single individual, the phenotype was only observed in progeny generations whose parental generation had some incidence of the phenotype (Table S3). Currently, we cannot rule out that the overall rarity of the MSH1-dr phenotype by the F_4_ generation may be the consequence of inadvertent selection rather than a natural tendency to gradually stabilize away from the phenotype.

To ensure that the observed variation was not the consequence of inadvertent seed contamination or outcrossing, 50 SSR markers were used to test a number of derived lines, which produced no evidence of polymorphism (Figure S3; Table S4). This analysis was extended with 1778 SNP markers that, when assayed across five different MSH1 F_2_ individuals and the wild type Tx430, detected less than 0.8% variation (Table S5, Figure S4). In Arabidopsis, the *msh1* mutant genome was DNA sequenced, with genome alignment and *de novo* assembly producing no evidence of unexplained genome rearrangement or unusual mutation frequency (unpublished). These data, together with reproducibility of the phenomenon, argue against the developmental reprogramming phenotype as a consequence of genome hypermutability.

### Significant increases in trait values persist for multiple generations

**Figure 3:**
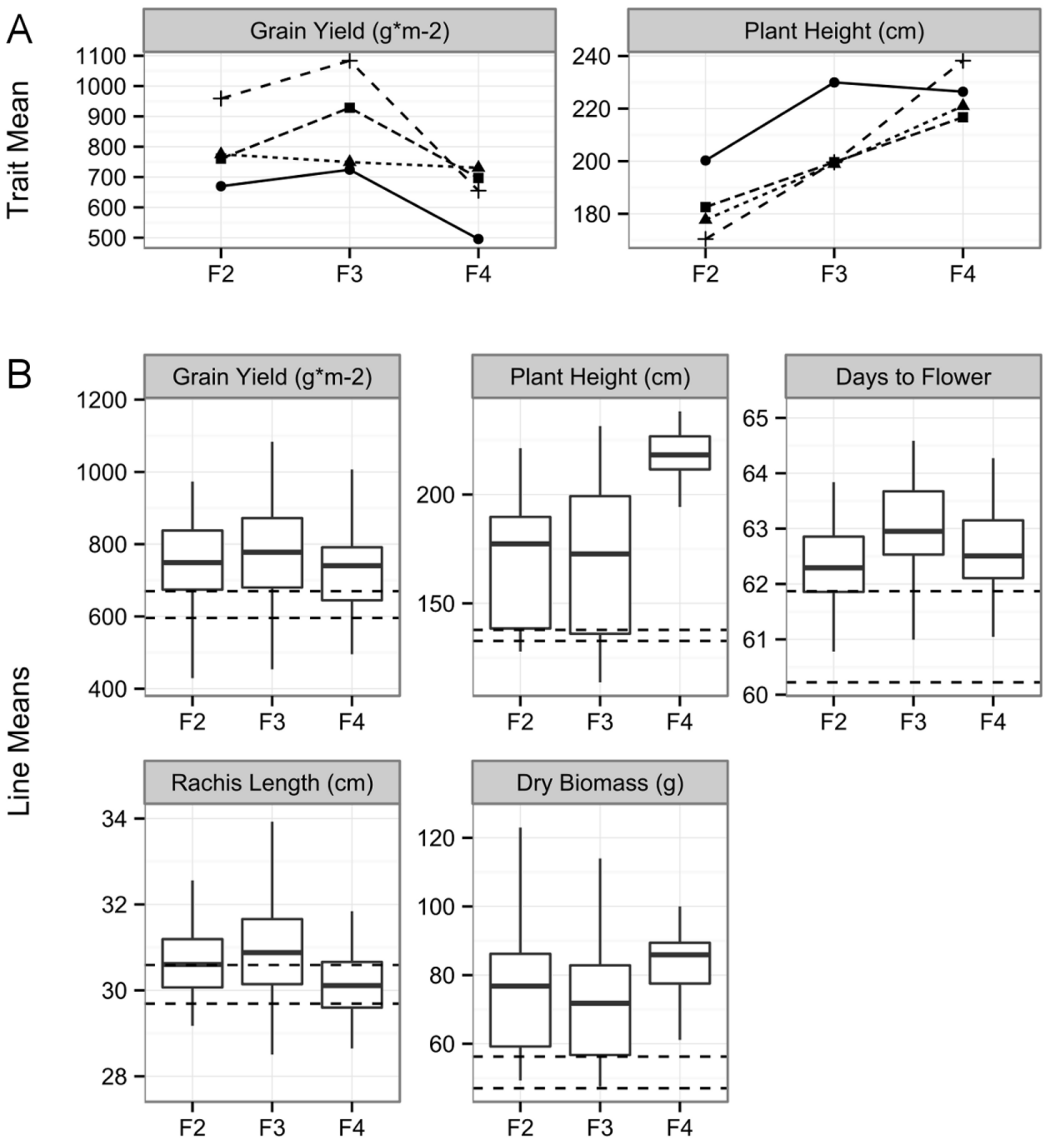
Phenotypic changes over MSH1 F2, F3 and F4 generations. (A) Selection had varying results, with response for yield into the F_3_ generation, but not into the F_4_ generation. For each lineage, the mean generation performance is represented as a point. (B) Boxplots of F_2_, F_3_, and F_4_ line means for various traits, giving a population-wide view of line performance. Dashed lines indicate the 95% confidence interval for wild type Tx430 mean.

From the MSH1 F_2_ families, individuals were self-pollinated and selected for grain yield and plant height to the F_3_ and F_4_ generations. F_4_ lines, along with F_3_ and F_2_ lines from remnant seed, were evaluated together in a 2011 field experiment. Despite weak selection intensity (33% and 38% of phenotyped plants were propagated to F_3_ and F_4_, respectively, based on grain yield), derived F_3_ and F_4_ lines showed differences in grain yield and plant height, as well as differences in dry biomass and panicle length (Figure 3, Figure S2, Table S6). Differences were detectable even when F_3_ and F_4_ lines were analyzed separately or when a model term for generation was included, indicating that the variation did not simply come from maternal effects. While some traits appeared to be correlated, such as flowering time and grain yield, no correlation was detected between plant height and grain yield, indicating that height was not pleiotropically affecting grain yield (Figure S5).

Although the F_3_ generation showed higher variance for some traits compared to the F_2_ generation, for all measured traits the F_4_ generation showed lower variance compared to the F_2_ generation (Figure 2A). Furthermore, in contrast to the F_2_ generation, we did not find significant heterogeneity for variance in grain yield among wild type, F_3_ and F_4_ lines (*p*>0.1, Brown-Forsythe test; p<0.01 among F_2_ lines and wild type).

Analysis of several direct lineages from F_2_ to F_4_ showed high response to selection for plant height but variable response for grain yield (Figures 3A, S6). Overall, gains in the F_4_ were more modest compared to the F_3_, implying progress may taper off by F_4_ in self-pollinated lineages. Indeed, there is evidence that the F_3_ generation may be the most vigorous. As a population, it appears to have slightly higher overall grain yield than the F_2_ or F_4_. Nevertheless, the population mean for grain yield in the F_4_ remains higher than that of wild type Tx430 (Figure 3B).

### Line×environment interactions suggest an additional component to G×E

**Figure 4:**
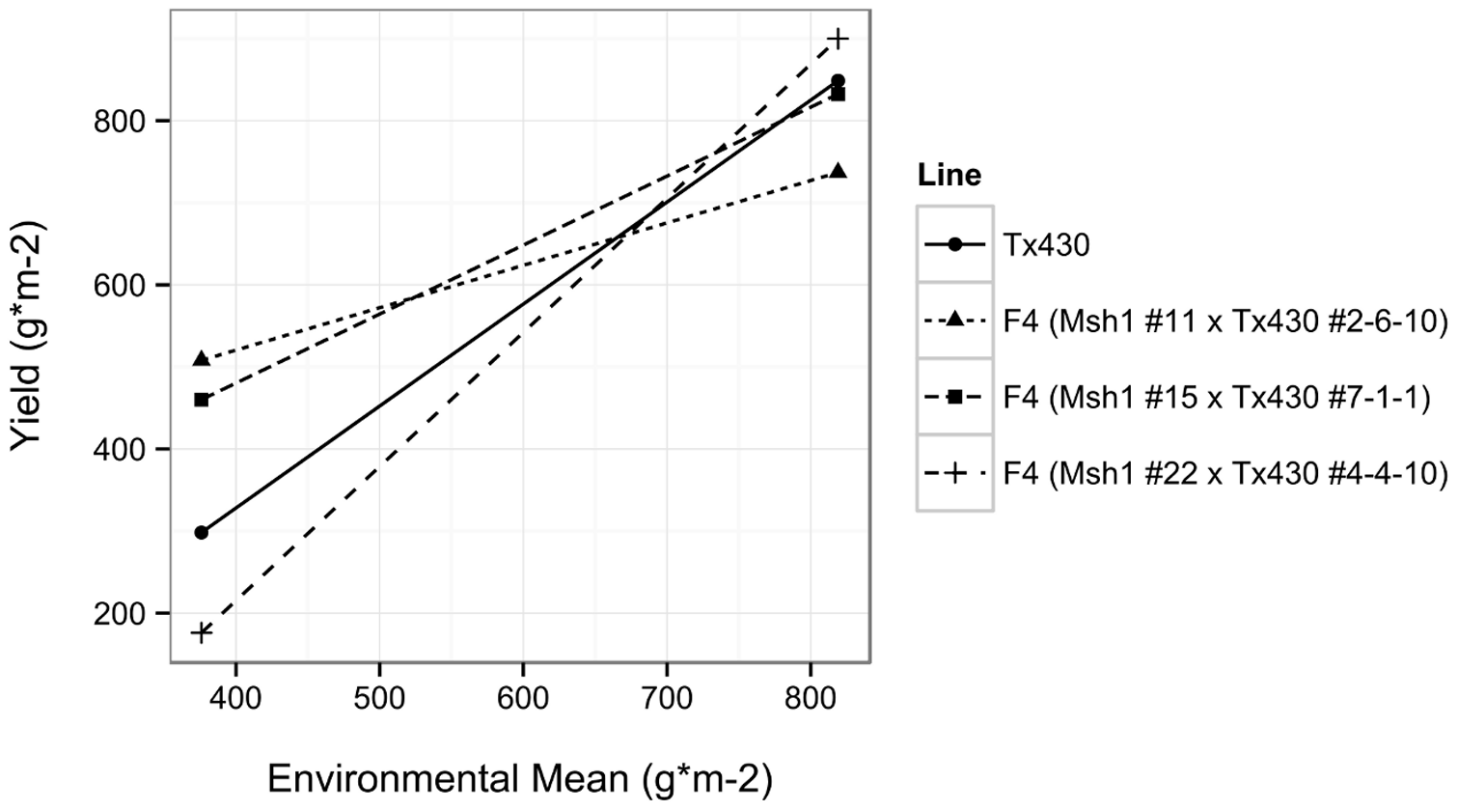
Line performance shows environmental interactions. Joint regression (with Wright modification) indicates differential response between lines to location.

As plant development is heavily influenced by the surrounding conditions, genotype×environment interactions (G×E) have major impacts on phenotype. The causes underlying G×E effects can potentially come from multiple sources, both genetic and non-genetic [19]. We evaluated the yield performance of three F_5_ families alongside wild type Tx430 at two different locations, which displayed a large difference in environmental means. Although the lines showed little between-line difference at the site of the earlier experiments (which may be a consequence of year-to-year climate effects), they showed large differences at the second site, which was more drought-stressed, demonstrating a line×environment effect (Figures 4, S7; Table S7). Results at the first site also suggest that, depending on conditions, variation in these materials could begin to dissipate at around the F_5_ generation. The outcomes of these experiments indicate that plant materials with little to no genetic variation have the potential to exhibit substantial variation in response to environmental influences, which may reflect epigenetic×environmental interactions.

## Discussion

A substantial range of sorghum phenotypic variation observed in this study appears to be primarily non-genetic, and is induced by crossing to a MSH1-dr line, altered through *MSH1* suppression in a previous generation. The MSH1-dr lines used in this study were maintained as transgene-nulls seven generations following segregation of the transgene, suggesting that the non-genetic properties of the MSH1-dr line are stable through multiple rounds of self-pollination [14]. We do not presume that all of the variation observed is non-genetic; the observed bimodal distribution for plant height could support an alternative hypothesis of markedly enhanced reversion frequency for the dwarfing gene, *dw3*, in line Tx430 [20]. If this is the case, the unusually high reversion rate may be the consequence of increased local recombination, possibly due to cytosine methylation redistribution [21], [22]. We are investigating this possibility presently. Nevertheless, we see additional height variation within short and tall plants, indicating variation beyond a single-locus.

The range of phenotypic variation observed is surprising. While we were not able to take measurements of all parameters for this initial study, the F_3_ and the F_4_ generations showed highly significant increases in above-ground biomass and grain yield over Tx430 wild type. One interpretation of these increases would be that *dw3* reversion could cause pleiotropic changes in plant architecture. However, the greater range of plant height, panicle architecture and yield variation observed in this study appears to exclude that possibility [23].

The observation of line×environment interaction in test plots suggests that at least some portion of the genotype×environment interaction that is commonly observed in varietal studies may be non-genetic, which is supported by other recent studies [24]. The *MSH1* system may be useful in understanding this type of environmental influence and selecting for enhanced stability of crop performance.

MSH1-dr transgene null lines developed on elite inbred genetic backgrounds would permit direct incorporation of the MSH1-enhanced growth phenomenon to hybrid production. However, studies to date have not observed the greatest gain in growth to occur in the derived F_1_ populations, suggesting that the effects we observe in this system may be distinct from heterosis. It is possible that self- or open-pollination breeding will prove more effective at capturing maximal growth gain derived from *MSH1* manipulation. The transgene-null MSH1-dr line crossed to its wild type counterpart produces maximum variation in the F_2_ population, at which point selection appears to be most effective. Large-scale seed increase in F_3_ and F_4_ generations permits rapid capture of the growth enhancement as variation tapers off. In our experience with this system, variation observed in the F_2_ population tends to produce above wild type performance more often than below (Figure 3B). Consequently, development of MSH1-dr in an elite line followed by selection in the F_2_, appears to result in, by the F_4_, a population that is uniform genetically yet enhanced in growth vigor and productivity.

The progress, response to selection, and final phenotypic outcomes observed in this study are of sufficient magnitude to suggest that untapped non-genetic potential resides within crops. One possibility is that epigenetic changes such as DNA methylation may either directly cause or are indicators of such variation. In Arabidopsis, mutation of genes that comprise the DNA methylation machinery, followed by crossing to wild type for development of recombinant inbred lines, has provided valuable information on the phenotypic consequences of epigenomic perturbation, as well as heritability and stability of epigenetic changes [25], [26]. It has been suggested that doubled haploids, subjected to recursive selection for mitochondrial behavior, can produce epigenetic variation that may be amenable to selection [27]. Somaclonal variation derived from plant tissue culture has also been associated with epigenetic changes [28]. Whether crop enhancement using *MSH1* manipulation will produce crop vulnerabilities not yet considered is under investigation. However, the performance of these plant materials under low rainfall conditions suggests that this methodology holds significant promise.

## Supporting Information

Figure S1 **F2 families generally exhibit wider distributions for panicle weight.** The majority of F2 families have an altered distribution of values for panicle weight, leading to generally flatter distributions with longer tails compared to wild type Tx430, and indicating that a higher proportion of individuals have unusually large values. Data presented are from 2010 field planting.
(TIFF)

Figure S2 **F4 lines show trait differences compared to wild type.** Lines from the MSH1 F_4_ generation show differences between each other and compared to wild type Tx430. Points represent line means; error bars represent 95% confidence intervals, which are shorter in wild type Tx430 due to higher sample size.
(TIFF)

Figure S3 **Sample SSR marker analysis.** Sorghum genomic DNAs were prepared from wild type Tx430, Tx430 MSH1-DR line (transgene-null, displaying the dwarfed, tillered, delayed flowering phenotype), one F_2_ and seven F_4_ lines selected for phenotypic diversity. The sweet sorghum variety Wray was included as a control. The SSR marker shown is generated with SAM16073 primers. Arrow shows detected DNA polymorphism. M designates marker lane, with fragment sizes (bp) shown at left. The 1500 and 35 bp fragments are internal markers used to calibrate each lane.
(TIFF)

Figure S4 **Sorghum genetic map with markers displaying heterozygous genotype.**
(TIFF)

Figure S5 **No correlation was found between plant height and grain yield.** Each point represents a line mean. Spearman's rank correlation coefficient = 0.02 (p-value = 0.83).
(TIFF)

Figure S6 **Shifts in phenotypic means by individual lineages.** Several lineages from F2 to F4 were (re)planted within the same field experiment and measured for (A) yield per panicle and (B) plant height, with varying results in terms of response to selection. Representative lineages from independent crosses are shown.
(TIFF)

Figure S7 **Line×environment effects were detected from a multiple location experiment.** Although three F_4_ lines (bulked from indicated F3 parent) were similar to wild type Tx430 in grain yield when grown in one location (Havelock), significant differences emerged when grown another location with a more challenging environment (Mead). Data were collected from a field experiment in 2012.
(TIFF)

Table S1 **MSH1-dr×Tx430 derived populations show more variation compared to wild type Tx430.** Data were acquired from 2011 field experiment. Brown-Forsythe tests for homogeneous variances were performed between all individuals of indicated generation versus wild type (e.g. all F_2_ vs Tx430, all F_3_ vs Tx430).
(DOCX)

Table S2 **Frequency of MSH1-dr phenotype (8.4%) in F3 families derived from sorghum MSH1-dr×Tx430.** Data were acquired from plants grown in greenhouse conditions.
(DOCX)

Table S3 **Msh1-dr phenotype shows a partially heritable or metastable component.** From each of ten lines, a single individual that did not display the MSH1-dr phenotype was grown along with its parental generation. Parental and progeny generation frequencies were then counted with N≥105 in each generation.
(DOCX)

Table S4 **SSR marker polymorphism data for 43 markers.** Markers were scored as + or − relative the pattern of Tx430 wild type. SSR markers were selected based on their polymorphic behavior in comparisons of Tx430 and a sweet sorghum variety, Wray. Assays included a transgene-null Tx430 line displaying the developmental reprogramming phenotype (DR), one F_2_, two F_3_, and seven F_4_ lines.
(DOCX)

Table S5 **SNP marker analysis.** (A) Summary of Het %. (B) A list of all the markers with heterozygous genotype. Markers with heterozygous genotypes are ordered by chromosome and genetic distance. The remainder (not shown) had homozygous genotypes. Marker genotypes of the six lines are similar but for the two markers highlighted in yellow. Markers showing a heterozygous genotype represent the true heterozygous genotype, not heterogeneity at the markers since only a single plant was sampled for DNA.
(DOCX)

Table S6 **Line effects on trait values are significant.** Data for each trait listed below were fit to a linear mixed model, with results indicating differences between lines. Line was treated as a fixed effect while block and replicate were treated as random effects. Separately analyzing lines by generation or general height class, or adding a model term for generation and height class, did not affect conclusions. The models were used to estimate trait means and confidence intervals (Figure 3B, S1).
(DOCX)

Table S7 **Analysis for significant effects using a mixed model indicates that line, location, and line×location are all significant.** See methods for model; sample size N = 121.
(DOCX)
