# Supplementary figures and images for: MSH1-Induced Non-Genetic Variation Provides a Source of Phenotypic Diversity in Sorghum bicolor
Source: PLoS One. 2014 Oct 27;9(10):e108407. doi: 10.1371/journal.pone.0108407 (PMC4209972; doi:10.1371/journal.pone.0108407)

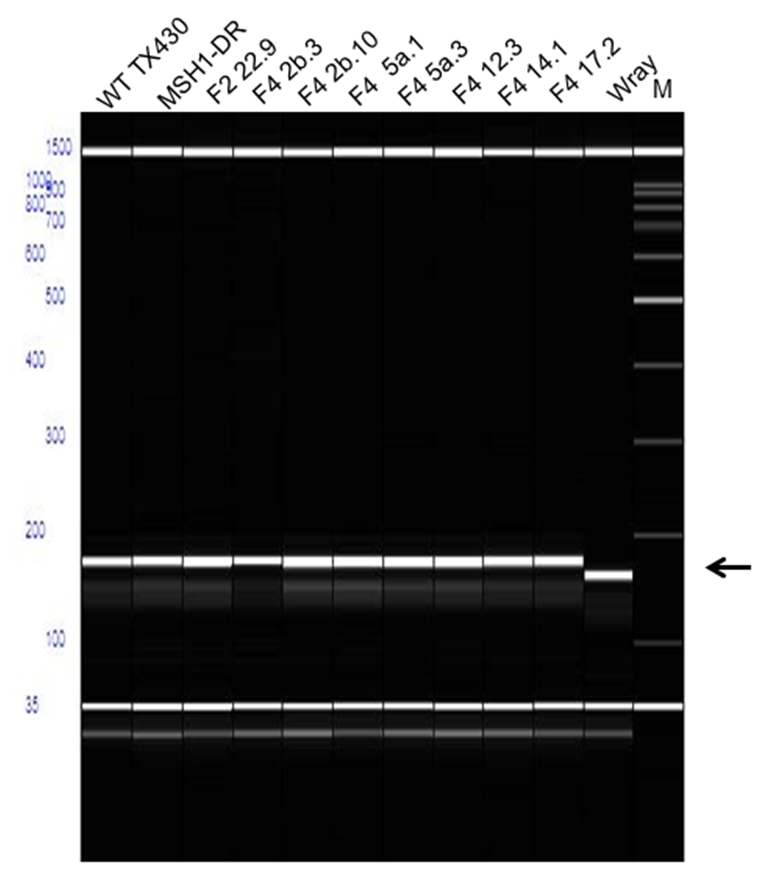

Supplement: Figure S3 — Sample SSR marker analysis. Sorghum genomic DNAs were prepared from wild type Tx430, Tx430 MSH1-DR line (transgene-null, displaying the dwarfed, tillered, delayed flowering phenotype), one F2 and seven F4 lines selected for phenotypic diversity. The sweet sorghum variety Wray was included as a control. The SSR marker shown is generated with SAM16073 primers. Arrow shows detected DNA polymorphism. M designates marker lane, with fragment sizes (bp) shown at left. The 1500 and 35 bp fragments are internal markers used to calibrate each lane. (TIFF) [file pone.0108407.s003.tiff]

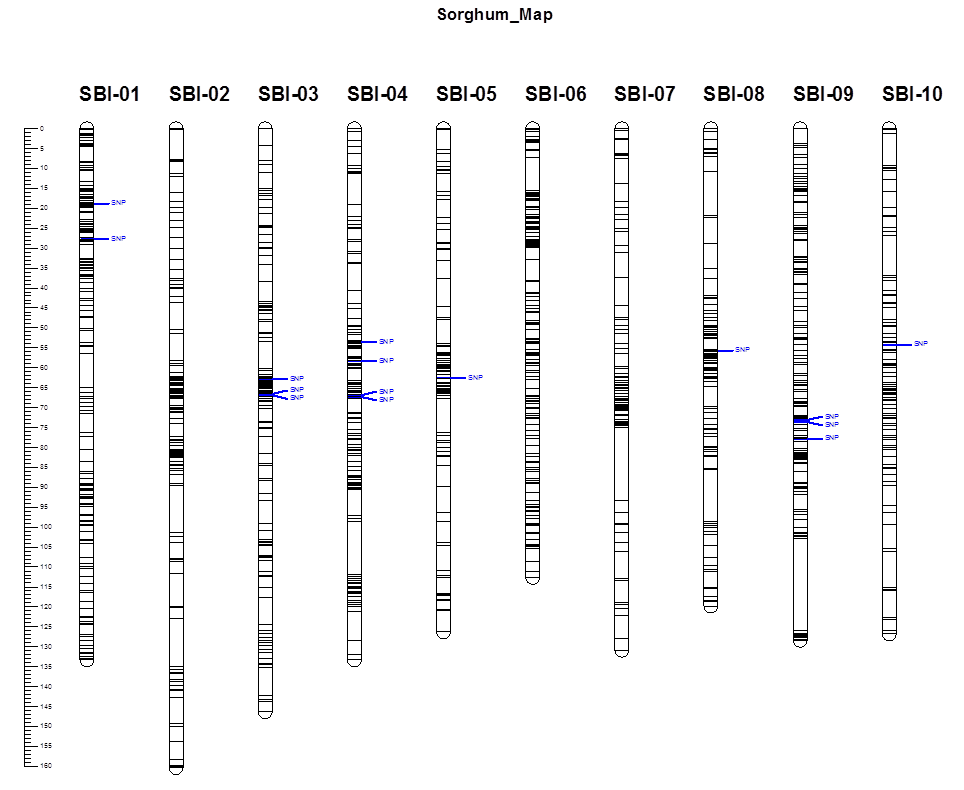

Supplement: Figure S4 — Sorghum genetic map with markers displaying heterozygous genotype. (TIFF) [file pone.0108407.s004.tiff]
