# Supplementary material for: MSH1-Induced Non-Genetic Variation Provides a Source of Phenotypic Diversity in Sorghum bicolor
Source: PLoS One. 2014 Oct 27;9(10):e108407. doi: 10.1371/journal.pone.0108407 (PMC4209972; doi:10.1371/journal.pone.0108407)
Supplement: Table S1 — MSH1-dr×Tx430 derived populations show more variation compared to wild type Tx430. Data were acquired from 2011 field experiment. Brown-Forsythe tests for homogeneous variances were performed between all individuals of indicated generation versus wild type (e.g. all F2 vs Tx430, all F3 vs Tx430). (DOCX) [file pone.0108407.s008.docx]

**Table S1**

|  |  |  | **Brown-Forsythe test** | |
| --- | --- | --- | --- | --- |
| **Trait** | **Population** | **N** | **F-value** | **p-value** |
| Grain Yield (grams/m^2^) | Tx430 | 55 | -- | -- |
|  | F2 generation | 318 | 9.6194 | < 0.01 |
|  | F3 generation | 348 | 19.582 | < 0.001 |
|  | F4 generation | 235 | 6.9213 | < 0.01 |
| Plant Height (cm) | Tx430 | 192 | -- | -- |
|  | F2 generation | 1493 | 301.04 | < 0.001 |
|  | F3 generation | 1587 | 306.61 | < 0.001 |
|  | F4 generation | 947 | 79.396 | < 0.001 |
| Flowering Time (DAS) | Tx430 | 134 | -- | -- |
|  | F2 generation | 908 | 0.2843 | > 0.1 |
|  | F3 generation | 635 | 16.819 | < 0.001 |
|  | F4 generation | 524 | 3.0162 | < 0.1 |
| Rachis Length (cm) | Tx430 | 288 | -- | -- |
|  | F2 generation | 1980 | 0.1348 | > 0.1 |
|  | F3 generation | 2412 | 3.3373 | > 0.1 |
|  | F4 generation | 1404 | 0.328 | > 0.1 |
| Dry Biomass (grams/plant) | Tx430 | 23 | -- | -- |
|  | F2 generation | 163 | 17.137 | < 0.001 |
|  | F3 generation | 188 | 18.313 | < 0.001 |
|  | F4 generation | 116 | 7.2468 | < 0.01 |
