# Supplementary material for: MSH1-Induced Non-Genetic Variation Provides a Source of Phenotypic Diversity in Sorghum bicolor
Source: PLoS One. 2014 Oct 27;9(10):e108407. doi: 10.1371/journal.pone.0108407 (PMC4209972; doi:10.1371/journal.pone.0108407)
Supplement: Table S2 — Frequency of MSH1-dr phenotype (8.4%) in F3 families derived from sorghum MSH1-dr×Tx430. Data were acquired from plants grown in greenhouse conditions. (DOCX) [file pone.0108407.s009.docx]

**Table S2**

|  | **F3 family** | **Sample size** | **Mean plant height (cm)** | **# Individuals with wild type height or higher** | **# Individuals with dwarf height** |
| --- | --- | --- | --- | --- | --- |
|  | 1 | 10 | 160 | 10 | 0 |
|  | 2 | 9 | 208 | 9 | 0 |
|  | 3 | 10 | 167 | 10 | 0 |
|  | 4 | 10 | 189 | 10 | 0 |
|  | 5 | 8 | 186 | 7 | 1 |
|  | 6 | 10 | 114 | 10 | 0 |
|  | 7 | 9 | 203 | 9 | 0 |
|  | 8 | 7 | 102 | 6 | 1 |
|  | 9 | 2 | 107 | 2 | 0 |
|  | 10 | 9 | 116 | 9 | 0 |
|  | 11 | 4 | 89 | 3 | 1 |
|  | 12 | 6 | 118 | 6 | 0 |
|  | 13 | 10 | 187 | 10 | 0 |
|  | 14 | 8 | 150 | 6 | 2 |
|  | 15 | 7 | 81 | 3 | 4 |
|  | 16 | 10 | 143 | 7 | 3 |
|  | 17 | 5 | 122 | 5 | 0 |
|  | 18 | 10 | 137 | 9 | 1 |
|  | 19 | 10 | 98 | 10 | 0 |
| **Total** | 19 | 154 | -- | 141 | 13 |
