# Supplementary material for: MSH1-Induced Non-Genetic Variation Provides a Source of Phenotypic Diversity in Sorghum bicolor
Source: PLoS One. 2014 Oct 27;9(10):e108407. doi: 10.1371/journal.pone.0108407 (PMC4209972; doi:10.1371/journal.pone.0108407)
Supplement: Table S3 — Msh1-dr phenotype shows a partially heritable or metastable component. From each of ten lines, a single individual that did not display the MSH1-dr phenotype was grown along with its parental generation. Parental and progeny generation frequencies were then counted with N≥105 in each generation. (DOCX) [file pone.0108407.s010.docx]

**Table S3**

|  | **Frequency of MSH1-dr phenotype** | | | |
| --- | --- | --- | --- | --- |
| **Line** | **Parental Frequency** | **%** | **Progeny Frequency** | **%** |
| Msh1...#11 x Tx430 #2-6-10-8 | 0/118 | 0% | 0/114 | 0% |
| Msh1...#11 x Tx430 #2-6-3-23 | 0/116 | 0% | 0/119 | 0% |
| Msh1...#15 x Tx430 #7-1-1-5 | 0/118 | 0% | 0/117 | 0% |
| Msh1...#15 x Tx430 #7-1-3-1 | 3/105 | 2.86% | 2/122 | 1.64% |
| Msh1...#15 x Tx430 #7-1-9-10 | 0/115 | 0% | 0/111 | 0% |
| Msh1...#22 x Tx430 #4-3-1-3 | 9/119 | 7.56% | 1/112 | 0.89% |
| Msh1...#22 x Tx430 #4-4-10-28 | 0/126 | 0% | 0/125 | 0% |
| Msh1...#24 x Tx430 #13-8-6 | 0/118 | 0% | 0/128 | 0% |
| Msh1...#24 x Tx430 #19-11-7 | 5/116 | 4.31% | 7/122 | 5.74% |
| Msh1...#28 x Tx430 #13-3-1 | 0/119 | 0% | 0/120 | 0% |
