# Supplementary material for: MSH1-Induced Non-Genetic Variation Provides a Source of Phenotypic Diversity in Sorghum bicolor
Source: PLoS One. 2014 Oct 27;9(10):e108407. doi: 10.1371/journal.pone.0108407 (PMC4209972; doi:10.1371/journal.pone.0108407)
Supplement: Table S4 — SSR marker polymorphism data for 43 markers. Markers were scored as + or − relative the pattern of Tx430 wild type. SSR markers were selected based on their polymorphic behavior in comparisons of Tx430 and a sweet sorghum variety, Wray. Assays included a transgene-null Tx430 line displaying the developmental reprogramming phenotype (DR), one F2, two F3, and seven F4 lines. (DOCX) [file pone.0108407.s011.docx]

**Table S4**

| **SSR Marker** | **Tx430** | **Tx430-DR** | **Wray** | **F2-22.9** | **F3-53** | **F3-68** | **F4-22.9** | **F4-2b.3** | **F4-2b.10** | **F4-5a.3** | **F4-12.3** | **F4- 14.1** | **F4- 17.2** |
| --- | --- | --- | --- | --- | --- | --- | --- | --- | --- | --- | --- | --- | --- |
| XCUP 1 | **-** | - | + | - | - | - | - | - | - | - | - | - | - |
| XCUP 5 | **-** | - | + | - | - | - | - | - | - | - | - | - | - |
| XCUP 26 | - | - | + | - | - | - | - | - | - | - | - | - | - |
| XCUP 28 | - | - | + | - | - | - | - | - | - | - | - | - | - |
| XCUP 32 | - | - | + | - | - | - | - | - | - | - | - | - | - |
| XCUP 48 | - | - | + | - | - | - | - | - | - | - | - | - | - |
| XCUP 50 | - | - | + | - | - | - | - | - | - | - | - | - | - |
| XCUP 61 | - | - | + | - | - | - | - | - | - | - | - | - | - |
| XCUP 69 | - | - | + | - | - | - | - | - | - | - | - | - | - |
| SAM 03605 | - | - | + | - | - | - | - | - | - | - | - | - | - |
| SAM 06337 | - | - | + | - | - | - | - | - | - | - | - | - | - |
| SAM 16073 | - | - | + | - | - | - | - | - | - | - | - | - | - |
| SAM 19028 | - | - | + | - | - | - | - | - | - | - | - | - | - |
| SAM 18581 | - | - | + | - | - | - | - | - | - | - | - | - | - |
| SAM 21112 | - | - | + | - | - | - | - | - | - | - | - | - | - |
| SAM 01312 | - | - | + | - | - | - | - | - | - | - | - | - | - |
| SAM 51414 | - | **-** | + | - | NA | NA | - | - | - | - | - | - | - |
| SAM 56359 | - | - | + | - | NA | NA | - | - | - | - | - | - | - |
| SAM 55010 | - | - | + | - | NA | NA | - | - | - | - | - | - | - |
| SAM 56942 | - | - | + | - | NA | NA | - | - | - | - | - | - | - |
| SAM 62005 | - | - | + | - | NA | NA | - | - | - | - | - | - | - |
| SAM 62186b | - | - | + | - | NA | NA | - | - | - | - | - | - | - |
| SAM 59974 | - | - | + | - | NA | NA | - | - | - | - | - | - | - |
| SAM 61376 | - | - | + | - | NA | NA | - | - | - | - | - | - | - |
| SAM 65125 | - | - | + | - | NA | NA | - | - | - | - | - | - | - |
| SAM 66160 | - | - | + | - | NA | NA | - | - | - | - | - | - | - |
| SAM 26858a | - | - | + | - | - | - | - | - | - | - | - | - | - |
| SAM 27170 | - | - | + | - | - | - | - | - | - | - | - | - | - |
| SAM 33545 | - | - | + | - | NA | NA | - | - | - | - | - | - | - |
| SAM 36890 | - | - | + | - | NA | NA | - | - | - | - | - | - | - |
| SAM 47801 | - | - | + | - | NA | NA | - | - | - | - | - | - | - |
| SAM 48589 | - | - | + | - | NA | NA | - | - | - | - | - | - | - |
| SAM 48870 | - | - | + | - | NA | NA | - | - | - | - | - | - | - |
| SAM 49411b | - | - | + | - | NA | NA | - | - | - | - | - | - | - |
| SAM 64056 | - | - | + | - | NA | NA | - | - | - | - | - | - | - |
| SAM 62693 | - | - | + | - | NA | NA | - | - | - | - | - | - | - |
| SAM 67633 | - | - | + | - | NA | NA | - | - | - | - | - | - | - |
| SAM 63126c | - | - | + | - | NA | NA | - | - | - | - | - | - | - |
| SAM 64809 | - | - | + | - | NA | NA | - | - | - | - | - | - | - |
| SAM 01952 | - | - | + | - | NA | NA | - | - | - | - | -- | - | - |
| SAM 40439 | - | - | + | - | NA | NA | - | - | - | - | - | - | - |
| SAM 42610 | - | - | + | - | NA | NA | - | - | - | - | - | - | - |
| SAM 39963 | - | - | + | - | NA | NA | - | - | - | - | - | - | - |

NA = not assayed
