# Supplementary material for: MSH1-Induced Non-Genetic Variation Provides a Source of Phenotypic Diversity in Sorghum bicolor
Source: PLoS One. 2014 Oct 27;9(10):e108407. doi: 10.1371/journal.pone.0108407 (PMC4209972; doi:10.1371/journal.pone.0108407)
Supplement: Table S5 — SNP marker analysis. (A) Summary of Het %. (B) A list of all the markers with heterozygous genotype. Markers with heterozygous genotypes are ordered by chromosome and genetic distance. The remainder (not shown) had homozygous genotypes. Marker genotypes of the six lines are similar but for the two markers highlighted in yellow. Markers showing a heterozygous genotype represent the true heterozygous genotype, not heterogeneity at the markers since only a single plant was sampled for DNA. (DOCX) [file pone.0108407.s012.docx]

**Table S5**

**A**

| **Pedigree** | **Total #markers** | **#Het** | **% Het** |
| --- | --- | --- | --- |
| (Msh111/Tx430):0002.0006.0010. | 1778 | 13 | 0.73% |
| (Msh115/Tx430):0007.0001.0001. | 1778 | 13 | 0.73% |
| (Msh115/Tx430):0008.0001.0002. | 1778 | 13 | 0.73% |
| (Msh122/Tx430):0004.0004.0010. | 1776 | 14 | 0.79% |
| (Msh124/Tx430):0019.0004. | 1774 | 13 | 0.73% |
| RTx430WT | 1773 | 14 | 0.79% |

**B**

| **Marker** | **Chr** | **Genetic Distance(cM)** | **Physical Distance (bp)** | **a1** | **a2** | **(MSH111/Tx430)** | **(MSH115/Tx430)** | **(MSH115/Tx430)** | **(MSHh122/Tx430)** | **(MSH124/Tx430)** | **RTx430WT** |
| --- | --- | --- | --- | --- | --- | --- | --- | --- | --- | --- | --- |
|  |  |  |  |  |  | **:0002.0006.0010.** | **:0007.0001.0001.** | **:0008.0001.0002** | **:0004.0004.0010.** | **:0019.0004.** |  |
| SNP1 | 1 | 18.8 | 19772012 | C | G | CG | CG | CG | CG | CG | CG |
| SNP2 | 1 | 27.7 | 31762071 | A | G | AG | AG | AG | AG | AG | AG |
| SNP3 | 3 | 62.9 | 33270511 | C | T | CT | CT | CT | CT | CT | CT |
| SNP4 | 3 | 66.6 | 42615046 | C | G | GG | GG | GG | CG | GG | GG |
| SNP5 | 3 | 66.9 | 35965148 | A | G | AG | AG | AG | AG | AG | AG |
| SNP6 | 4 | 53.6 | 13022975 | C | T | CT | CT | CT | CT | CT | CT |
| SNP7 | 4 | 58.4 | 23297070 | A | G | AG | AG | AG | AG | AG | AG |
| SNP8 | 4 | 66.9 | 41492135 | C | T | CT | CT | CT | CT | CT | CT |
| SNP9 | 4 | 67.3 | 42325806 | C | T | CT | CT | CT | CT | CT | CT |
| SNP10 | 5 | 62.6 | 16905084 | A | G | AG | AG | AG | AG | AG | AG |
| SNP11 | 8 | 55.7 | 12142806 | C | G | CC | CC | CC | CC | CC | CG |
| SNP12 | 9 | 73.1 | 43494421 | C | T | CT | CT | CT | CT | CT | CT |
| SNP13 | 9 | 73.6 | 43961814 | C | T | CT | CT | CT | CT | CT | CT |
| SNP14 | 9 | 77.8 | 46574813 | A | G | AG | AG | AG | AG | AG | AG |
| SNP15 | 10 | 54.2 | 10653756 | G | T | GT | GT | GT | GT | GT | GT |
