# Supplementary material for: MSH1-Induced Non-Genetic Variation Provides a Source of Phenotypic Diversity in Sorghum bicolor
Source: PLoS One. 2014 Oct 27;9(10):e108407. doi: 10.1371/journal.pone.0108407 (PMC4209972; doi:10.1371/journal.pone.0108407)
Supplement: Table S6 — Line effects on trait values are significant. Data for each trait listed below were fit to a linear mixed model, with results indicating differences between lines. Line was treated as a fixed effect while block and replicate were treated as random effects. Separately analyzing lines by generation or general height class, or adding a model term for generation and height class, did not affect conclusions. The models were used to estimate trait means and confidence intervals (Figure 3B, S1). (DOCX) [file pone.0108407.s013.docx]

**Table S6**

| **Population** | **Response variable of model** | **df** | **Sample size** | **F-statistic of**  **Line effect** | **p-value of**  **Line effect** |
| --- | --- | --- | --- | --- | --- |
| All lines | Grain Yield (g/m^2^) * | 160 | 956 | 4.985 | < 0.001 |
|  | Plant height (cm) | 167 | 3537 | 159.6 | < 0.001 |
|  | Flowering time (DAS) * | 136 | 1787 | 10.27 | < 0.001 |
|  | Rachis Length (cm) * | 165 | 3495 | 5.06 | < 0.001 |
|  | Dry Biomass (g/plant) * | 167 | 490 | 5.1 | < 0.001 |

* Box-cox transformed for model fit.
