# Supplementary material for: MSH1-Induced Non-Genetic Variation Provides a Source of Phenotypic Diversity in Sorghum bicolor
Source: PLoS One. 2014 Oct 27;9(10):e108407. doi: 10.1371/journal.pone.0108407 (PMC4209972; doi:10.1371/journal.pone.0108407)
Supplement: Table S7 — Analysis for significant effects using a mixed model indicates that line, location, and line×location are all significant. See methods for model; sample size N = 121. (DOCX) [file pone.0108407.s014.docx]

**Table S7**

| **Fixed Effect** | **df** | **Denom** | **F-statistic** | **p-value** |
| --- | --- | --- | --- | --- |
| Line | 3 | 87.831 | 3.5988 | < 0.05 |
| Location | 1 | 14.136 | 175.5053 | < 0.001 |
| Line x Location | 3 | 87.831 | 16.2472 | < 0.001 |
